# Effects of μ-Agatoxin-Aa1a in Cellular and Rat Models of Myocardial Ischemia–Reperfusion Injury

**DOI:** 10.3390/ijms27209287

**Published:** 2026-10-19

**Authors:** Elena Iurova, Dinara Dolgova, Natalya Mikheeva, Anna Ermakova, Dmitrii Sugak, Aigul Gilmutdinova, Aleksandr Fomin, Yury Saenko

**Affiliations:** 1 Laboratory of Research and Development of Peptide Drugs and Vaccines, S. P. Kapitsa Research Institute of Technology, Ulyanovsk State University, Ulyanovsk 432017, Russia; urovaev523@gmail.com (E.I.); tseplaiaaa@stud.ulsu.ru (A.E.); dmitriysugak@mail.ru (D.S.); gilmutdinovaajjgul@gmail.com (A.G.); mr.fominan@yandex.ru (A.F.); 2 Laboratory of Molecular and Cellular Biology, S. P. Kapitsa Research Institute of Technology, Ulyanovsk State University, Ulyanovsk 432017, Russia; dolgova.dinara@yandex.ru (D.D.); tcyganovana@mail.ru (N.M.); 3 Department of Physiology and Pathophysiology, Ulyanovsk State University, Ulyanovsk 432017, Russia; 4 Department of Biology, Ecology and Environmental Management, Ulyanovsk State University, Ulyanovsk 432017, Russia; 5 Autonomous Non-Commercial Organization “Agency for Scientific and Technical Initiatives”, Ulyanovsk Ave. 13, Ulyanovsk 432067, Russia

**Keywords:** ischemia, reperfusion, cardiomyocytes, toxin, sodium channel

## Abstract

Myocardial infarction is one of the common cardiovascular diseases. Sodium overload in the myocardium is another of the most important mechanisms underlying ischemia/reperfusion injury. Na^+^ entry into the cell through ion channels can be controlled using peptide toxins targeting ion channels. In our work, we analyze the use of mu-agatoxin-Aa1a, as a sodium channel modulator, as a cytoprotective agent in ischemia/reperfusion injury. For IRI modeling in vitro, the H9C2 culture was placed under oxygen-glucose deprivation conditions followed by reperfusion. Cell death and ion dynamics were recorded using a fluorescent plate reader. Reversible myocardial ischemia of rats in vivo was created by applying an occluder under the main branch of the left coronary artery. Histological and biochemical changes were recorded. The study demonstrated the ability of mu-agatoxin-Aa1a myocardial cells to adapt to rapidly changing extracellular conditions upon restoration of normal blood flow after a period of ischemia. Thus, the study allows us to consider mu-agatoxin-Aa1a as a biologically active molecule with a targeted effect to enhance the adaptation of cardiomyocytes to OGD/reperfusion conditions.

## 1. Introduction

Myocardial infarction is one of the most common cardiovascular diseases in the world. The cause of infarction is the complete or partial cessation of blood supply to cardiomyocytes in a part of the myocardium [1]. Cardiomyocytes are strictly oxygen-dependent because the heart requires an extensive energy supply to maintain its function, which is provided by adenosine triphosphate (ATP) derived from oxidative phosphorylation [2]. Interruption of coronary blood flow rapidly puts cardiomyocytes under stress and ultimately leads to cell death. Reperfusion reintroduces substrates for ATP and oxygen generation, providing normalization of pH and flushing out catabolic wastes accumulated during ischemia. These factors are paramount to myocardial tissue survival, but in cardiomyocytes after prolonged ischemia, these same factors can paradoxically exacerbate cellular damage, determining the transition from reversible to irreversible damage, resulting in ischemia/reperfusion injury (IRI) [3].

It has been known for a long time that sodium overload in the myocardium is the most important mechanism underlying ischemia/reperfusion injury [4]. After the onset of ischemia, the concentration of Na^+^ gradually increases. The massive Na^+^ influx into the cell, which continues during reperfusion by displacing excess H^+^ ions through the NHE exchanger, causes the sodium-calcium exchanger to work abnormally, pumping out Na^+^ in exchange for Ca^2+^ [5]. Temporary Ca^2+^ accumulation in the cytoplasm is sufficient to activate calcium-dependent lipases and proteases and a wide range of pathophysiological responses, including cardiomyocyte hypercontracture, mitochondrial dysfunction, osmotic overload, gap junction changes, and activation of inflammatory signaling [6], which leads to aggravation of myocardial damage. Under normal conditions, the steady (background) sodium ion current constitutes only a small fraction of the peak inward sodium current (*I_Na_*) recorded at the time of action potential generation [7]. However, it is significantly enhanced under hypoxic conditions and contributes significantly to the increase in Na+ over time [8]. The entry of Na^+^ into the cell through ion channels can be controlled by using ion channel blockers.

The strategy of using sodium channel blockers has been used in medical practice for a long time. For example, it is known that sodium channel blockers are used as membrane-stabilizing antiarrhythmic medications. These include medications such as quinidine, novocainamide, and ajmaline, etc., with moderate blockade of sodium channels, which is used in ventricular arrhythmias, atrial fibrillation, and tachycardia [9,10], as well as propafenone, ethacizine, flecainide, etc., with pronounced blockade of sodium channels and used in cases of arrhythmias, as well as in the post-infarction period [11]. These medications inhibit phase 0 (rapid depolarization) of the action potential in working myocardial cells of atria, ventricles, and the ventricular part of the cardiac conducting system. The electrophysiological peculiarity of these heart sections is that action potential phase 0 is driven by an inward sodium current into the cardiomyocytes. Partial blockade of potential-dependent sodium channels leads to slowing down of phase 0 of the action potential, which is accompanied by some slowdown of conduction in these heart sections. Despite the fact that this type of blocker has a pronounced effect on sodium ion concentration changes, there are few works devoted to the study of sodium channel blockers in IRI and all of them date back to 80–90s of the XX century [12,13]. However, the papers show a general trend that sodium channel blockers have a favorable effect on cardiomyocyte survival under ischemia/reperfusion conditions [14,15,16,17].

In our work, we consider mu-agatoxin-Aa1a toxin [Uniprot: T5G1A_AGEAP] from *Agelenopsis aperta* spider venom, which belongs to modulators of potential-dependent sodium channels, to reduce Na^+^ influx at the reperfusion stage and, therefore, to decrease IRI. However, modification of sodium channels by toxin may lead to increased sensitivity of these channels by shifting the activation curve to more negative potentials [18]. This leads to an increased probability of sodium channel opening. Conversely, such substances can act as ion channel inhibitors without affecting the kinetics of channel activation and inactivation, and bind to the channel in an inactivated state and stabilize it [19]. In addition, peptide toxins, to which mu-agatoxin-Aa1a belongs, by their nature have several distinctive properties, one of which is high selectivity towards a certain type of channels, due to which the desired effect can be achieved using small doses, in the order of nano- and micromolar ones. Another feature of mu-agatoxin-Aa1a is that it belongs to the knottins, a family of peptide toxins characterized by the presence of several cysteine residues that can form disulfide bridges, due to which they exhibit properties of increased stability with respect to a few parameters such as temperature, pH, and exposure to proteases, which significantly increases bioavailability.

Thus, the purpose of this work was to analyze the use of mu-agatoxin-Aa1a as a sodium channel modulator and cytoprotective agent in ischemia/reperfusion injury.

## 2. Results

**Figure 1:**
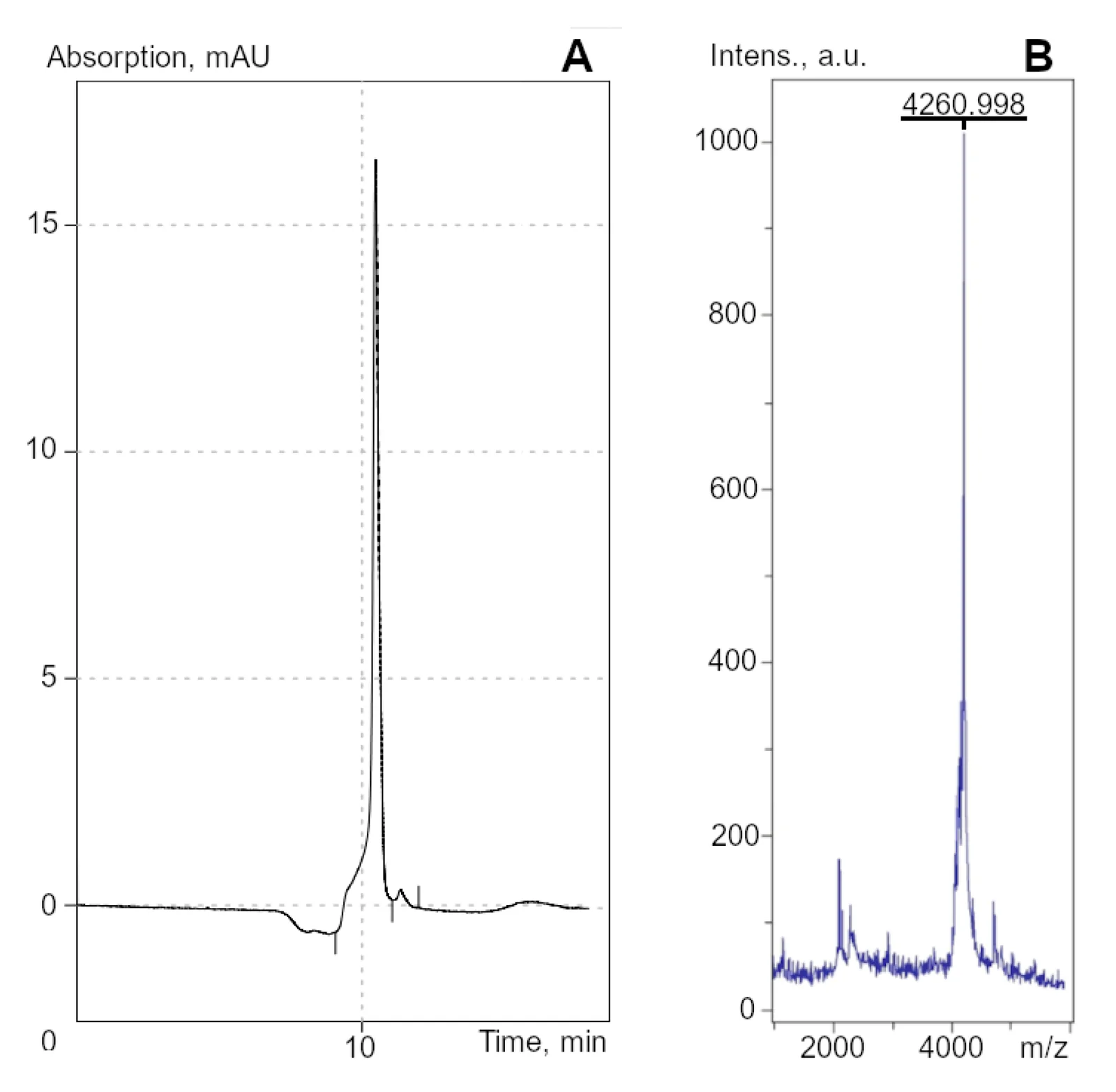
Chromatogram (**A**) and mass spectrogram (**B**) of mu-agatoxin-Aa1a toxin. The mass spectrogram confirms successful folding. Subsequent HPLC purification yielded a peptide purity of 98%.

Mu-agatoxin-Aa1a was prepared for the study using solid-phase peptide synthesis. The toxin was purified, and sulfide bond folding was performed. Finally, the desired structure of the toxin with purity of more than 90% was obtained (Figure 1).

**Figure 2:**
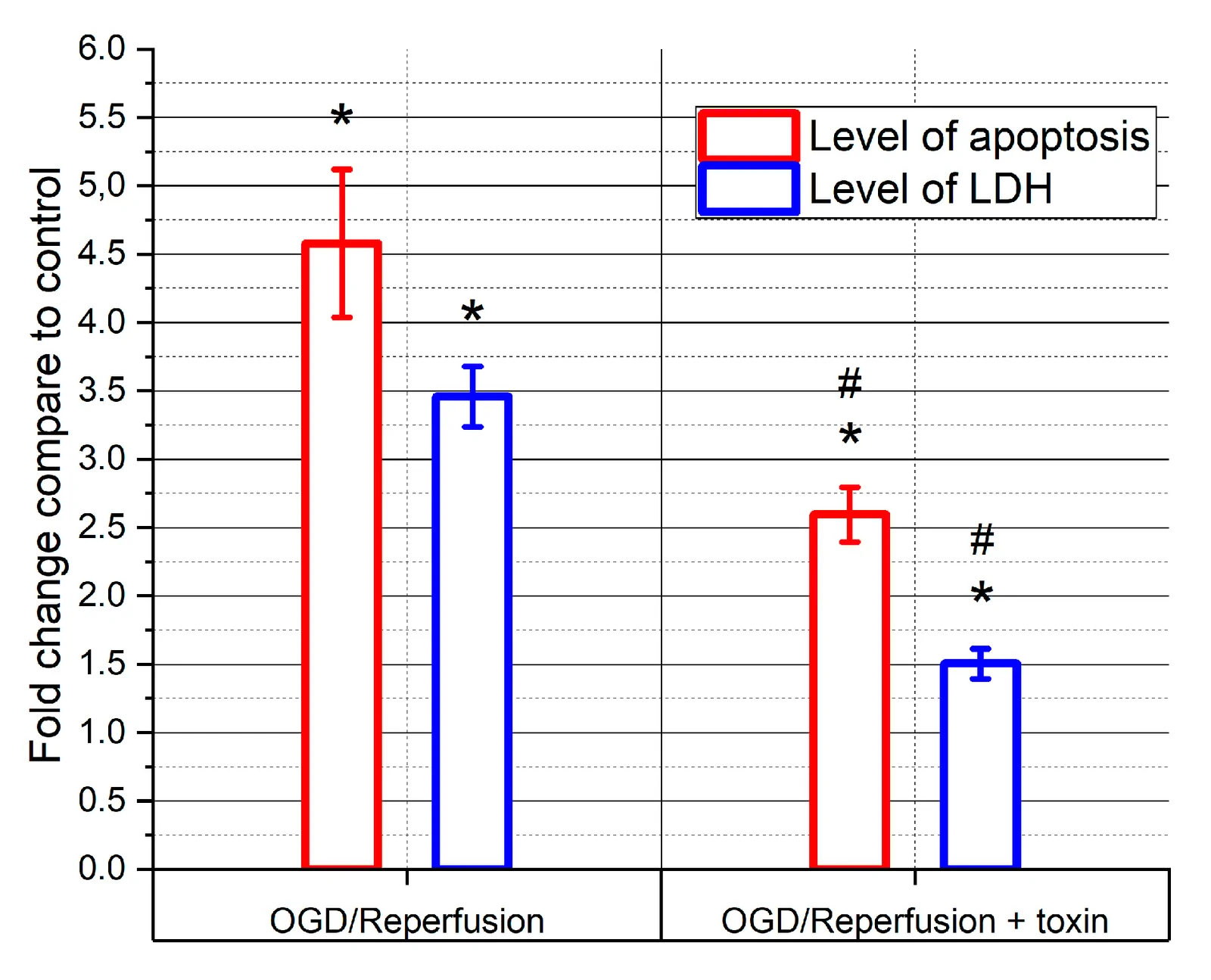
Apoptosis level and LDH concentration in H9C2 cardiomyocyte culture under ischemia/reperfusion (OGD/Reperfusion) conditions with mu-agatoxin-Aa1a at 50 nM concentration (the level of apoptosis was detected at the end point of reperfusion (2 h) using a fluorescent dye Yo-Pro 1 (1 μmol/L); LDH concentration was measured using a spectrophotometric method) (LDH reference values: 207–414 U/L; data are presented as experiment/control ratio (control = normal conditions) (*—difference from normal conditions, *p* < 0.02, #—difference from OGD/Reperfusion, *p* < 0.02).

When modeling IRI conditions, apoptosis developed in cardiomyocyte culture (Figure 2), and there was a significant increase in the LDH concentration, indicating membrane integrity disruption (necrosis). The toxin at a concentration of 50 nM reduced apoptosis by almost 40% and the LDH concentration by more than 2 times.

**Figure 3:**
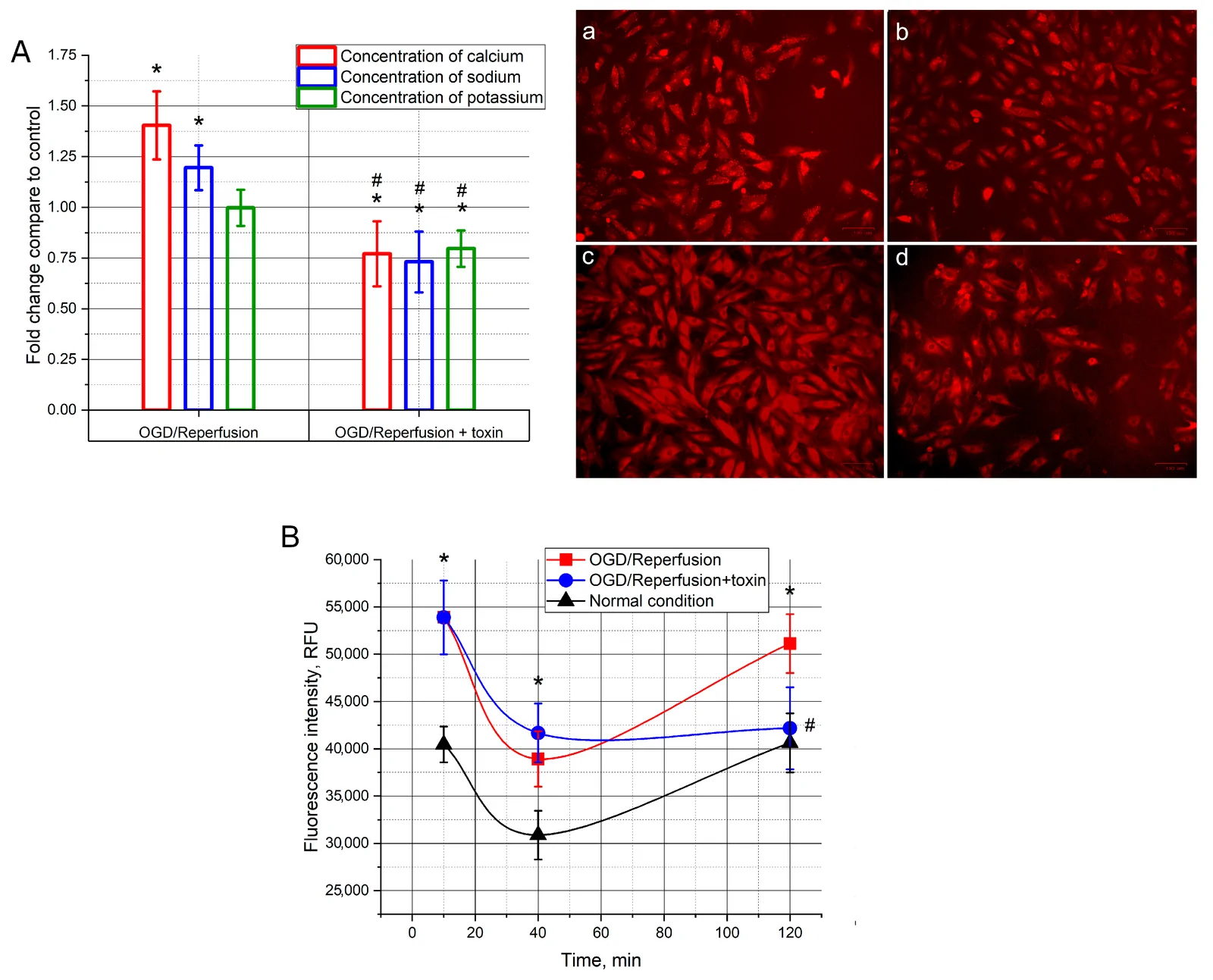
(**A**). Calcium, sodium, and potassium ion concentrations in H9C2 cardiomyocyte culture under ischemia/reperfusion (OGD/Reperfusion) conditions with mu-agatoxin-Aa1a at a concentration of 50 nmol/L (the concentration of calcium, sodium, and potassium ions was detected at the end point of reperfusion (2 h) using the corresponding fluorescent dyes: Rhod 2 AM for calcium, ION NATRIUM Green 2 AM for sodium, ION Potassium Green 2 AM for potassium at a final concentration of 500 nmol/L) (data are presented as experiment/control (normal conditions)) (*—difference from normal conditions, *p* < 0.02 #—difference from OGD/Reperfusion, *p* < 0.02). Color photo: Ca2+ ion concentration during Rhod 2 AM staining under OGD/Reperfusion conditions (**a**) and during toxin addition (**b**); Na+ ion concentration during ION NATRIUM Green 2 AM staining under OGD/Reperfusion conditions (**c**) and during toxin addition (**d**). (**B**). Dynamics of pH in H9C2 cardiomyocyte culture under ischemia/reperfusion (OGD/Reperfusion) conditions with mu-agatoxin-Aa1a at a concentration of 50 nmol/L (data are presented as experiment/control (normal conditions)) (*—difference from normal conditions, *p* < 0.02; #—difference from OGD/Reperfusion, *p* < 0.02).

Concentrations of calcium and sodium ions also underwent changes during IRI development (Figure 3A). Thus, there was an increased concentration of calcium and sodium, almost 1.3 and 1.2 times after 2 h reperfusion, while the concentration of potassium did not change. During the reperfusion phase, calcium concentrations decreased to almost half the experimental values and almost 20% below normal levels. Sodium concentrations also decreased by almost half, falling below normal levels. Potassium concentrations also decreased below normal levels.

One of the key parameters in the study was the examination of acid–alkali balance dynamics. Figure 3B shows that after ischemia, an increased pH level relative to normal conditions was observed in the first 10 min of reperfusion. During subsequent reperfusion, the pH level in the OGD/Reperfusion group decreased in the first 30 min, remaining elevated relative to normal conditions. After 2 h, the level increased again and remained above normal. In the group with toxin at a concentration of 50 nM, pH also decreased in the first 30 min from the onset of reperfusion, remaining elevated relative to normal conditions, but after 2 h it decreased to normal levels.

**Figure 4:**
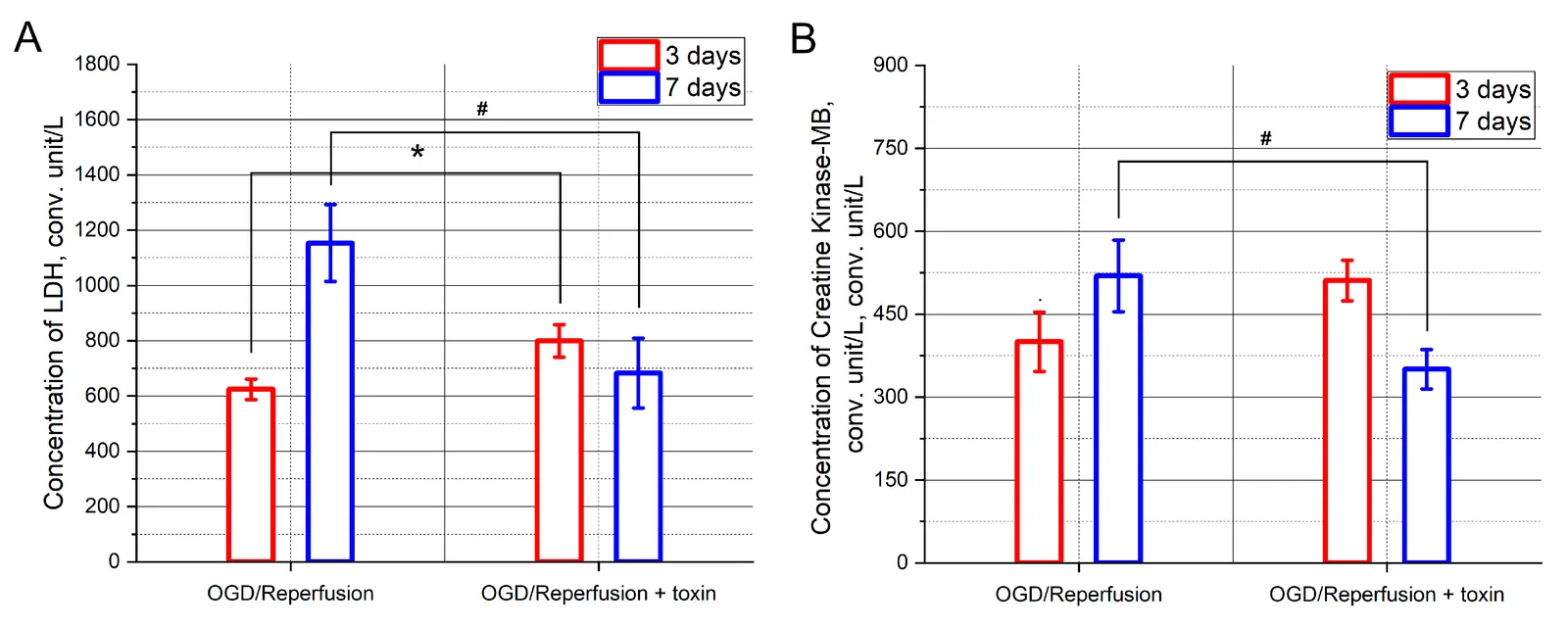
Activity of LDH (**A**) and creatine kinase-MB (**B**) in rat serum after myocardial infarction (*—difference from OGD/Reperfusion at 3 days, *p* < 0.02 (*n* = 3); #—difference from OGD/Reperfusion at 7 days, *p* < 0.02 (*n* = 6 for control, *n* = 7 for toxin)).

Changes in the activity of lactate dehydrogenase and creatinine kinase MB enzymes are shown in Figure 4.

An increase in LDH was noted in the OGD/Reperfusion group between days 3 and 7 from 624.67 ± 37.2 specific units/L to 1153.29 ± 138.95 specific units/L. In the group with the addition of the toxin, LDH decreased from 799.3 ± 58.7 specific units/L to 682.83 ± 125.9 specific units/L, which is almost 1.5 times lower than in the same group without the toxin. Creatinine kinase-MB also undergoes changes. Thus, after 7 days, the OGD/Reperfusion group showed a reading of 519.39 ± 65.07, while in the toxin-supplemented group, it was 350.45 ± 35.63 on day 7, which is also almost 1.5 times lower.

**Figure 5:**
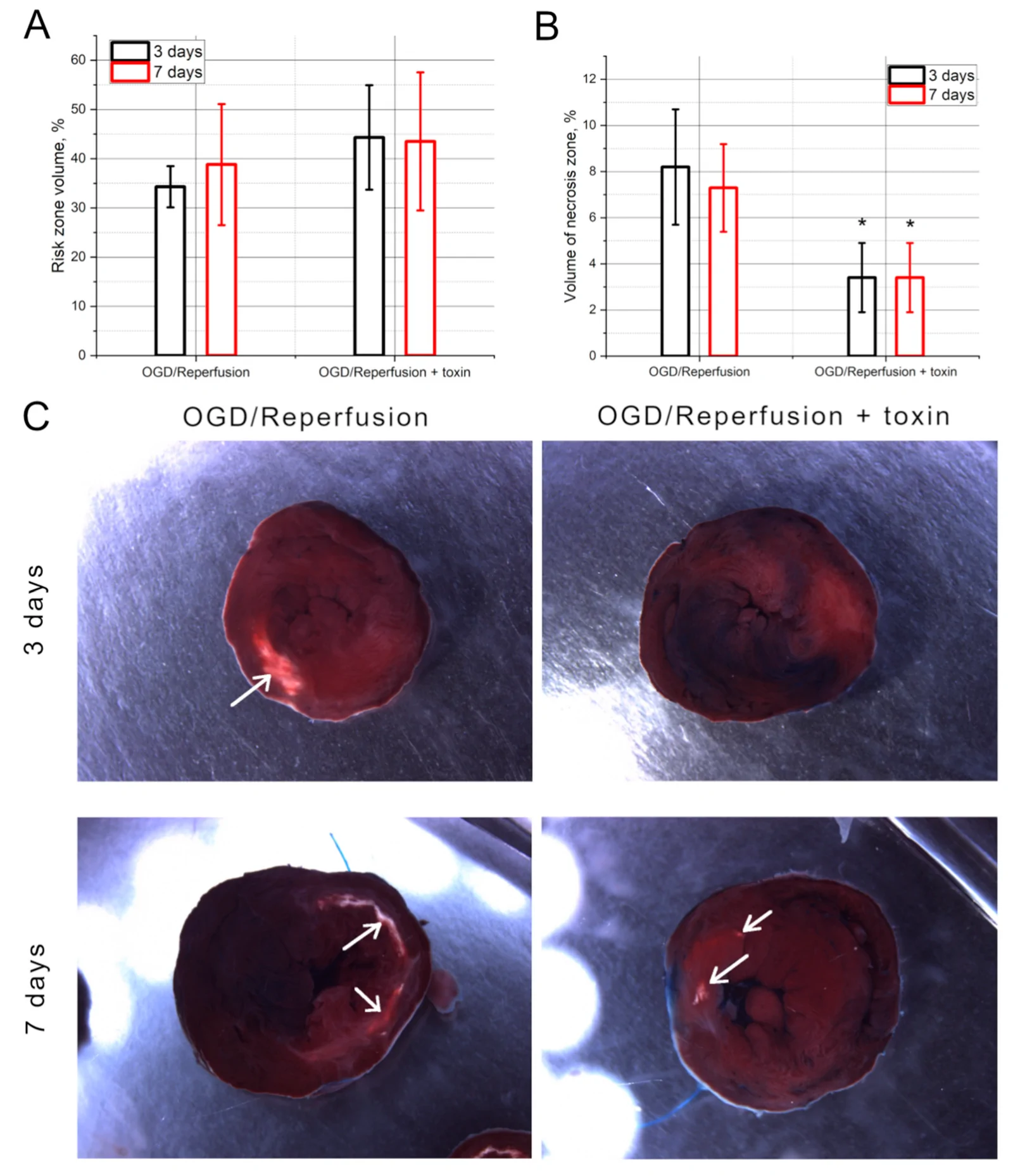
(**A**) Ratio of risk zones with Evans dye exclusion by cells and (**B**) necrotic zones with TTC exclusion (*—difference from the same group in OGD/Reperfusion, *p* < 0.02). (**C**). Representative images of necrotic zones in the OGD/Reperfusion groups with and without toxin after 3 (*n* = 3) and 7 days (*n* = 6 for control, *n* = 7 for toxin) (arrows indicate necrotic zones).

The change in the ratio of risk zones to necrotic zones is shown in Figure 5. In all groups, the risk zone remains unchanged over 7 days, while the necrotic zone decreases almost threefold in the toxin-added group by day 3 and maintains this value through day 7.

**Figure 6:**
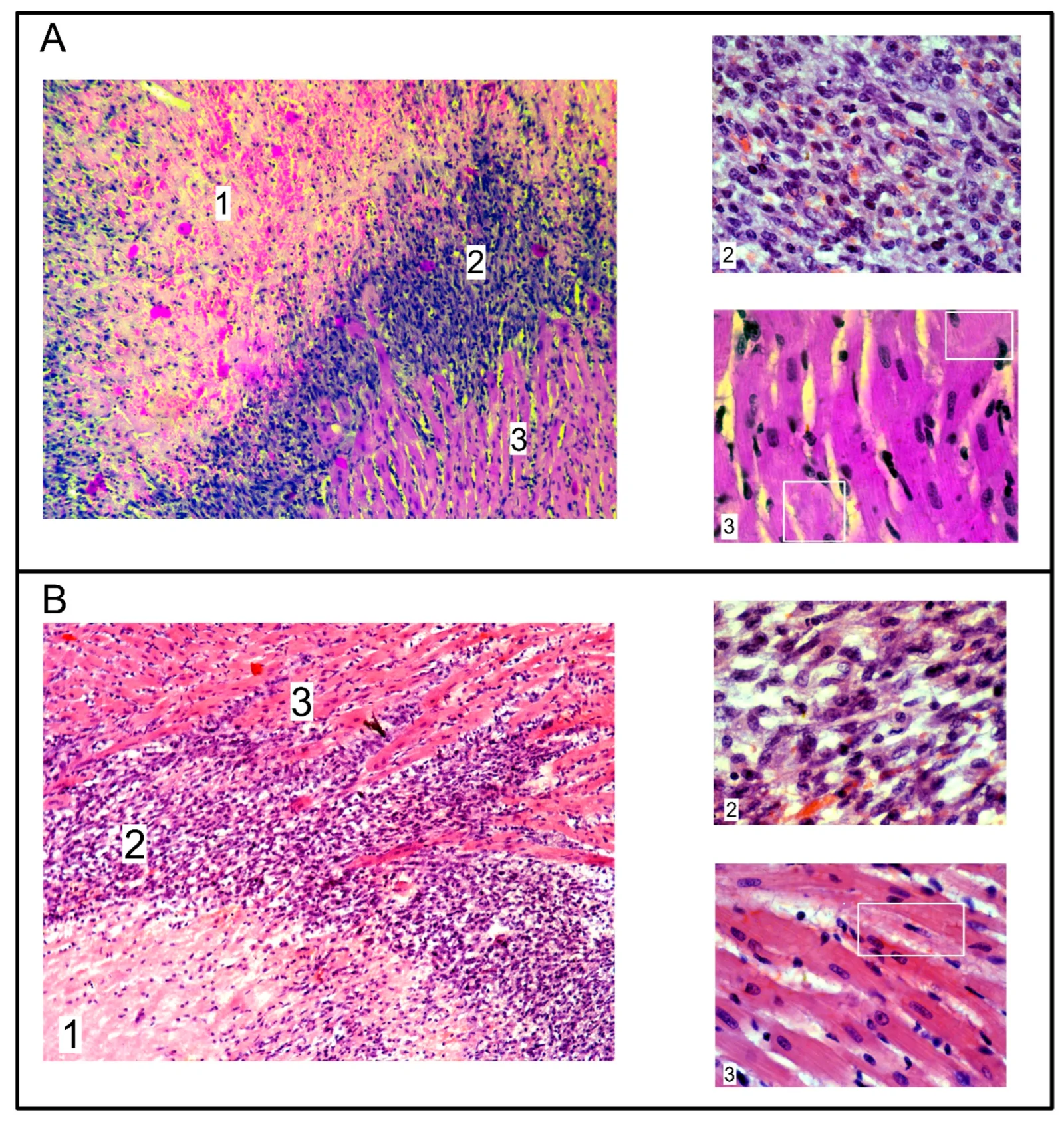
Rat myocardium after acute infarction on day 3 ((**A**)—OGD/Reperfusion; (**B**)—OGD/Reperfusion + toxin): 1—necrosis zone, 2—demarcation inflammation zone, 3—preserved myocardium (white frame—swollen cardiomyocytes). Overview photograph (**A**) shows a necrotic focus occupying a significant section area (1), cardiomyocytes with karyolysis and plasmolysis, and ischemic myocytes characterized by hypereosinophilia. The demarcation zone (2) is represented by polymorphonuclear leukocytes, macrophages, and a significant number of fibroblasts; vessels are thin walled, mostly empty. Preserved myocardium (3) is characterized by severe edema. Overview photograph (**B**) also shows a necrotic focus occupying a significant section area (1), with the formation of non-nuclear necrotic detritus. The demarcation zone (2) also includes polymorphonuclear leukocytes and fibroblasts, loosely arranged due to the greater number of hemocapillaries, which are mostly empty, as in (**A**) (2). The preserved myocardium (3) is also characterized by pronounced edema and pronounced transverse striation. Number of animals in the group: 3 days (*n* = 3), 7 days (*n* = 6 for control, *n* = 7 for toxin). Hematoxylin with eosin, × 100, × 600.

The described acute infarction in all experimental animals is transmural and involves the entire thickness of the myocardium. In animals of all experimental groups, by day 3, a large area of necrosis (coagulative type) with lysis of nuclei and homogenization of cardiomyocytes is identified. The extent of myocardial damage in animals of the experimental groups receiving the toxin is virtually identical to that in the control group (Figure 6A,B). The necrotic zone is separated from the functioning myocardium by a cellular wall representing a demarcation zone and consisting of neutrophils, macrophages, and lymphocytes. In the perifocal zone of ischemic damage in animals administered the toxin, signs of its organization are observed, manifested by the presence of fibroblasts, fine collagen fibers, and thin-walled capillaries. The preserved myocardium is characterized by interstitial edema, the transverse striation of cardiomyocytes is preserved, and the myocardial arteries are empty.

**Figure 7:**
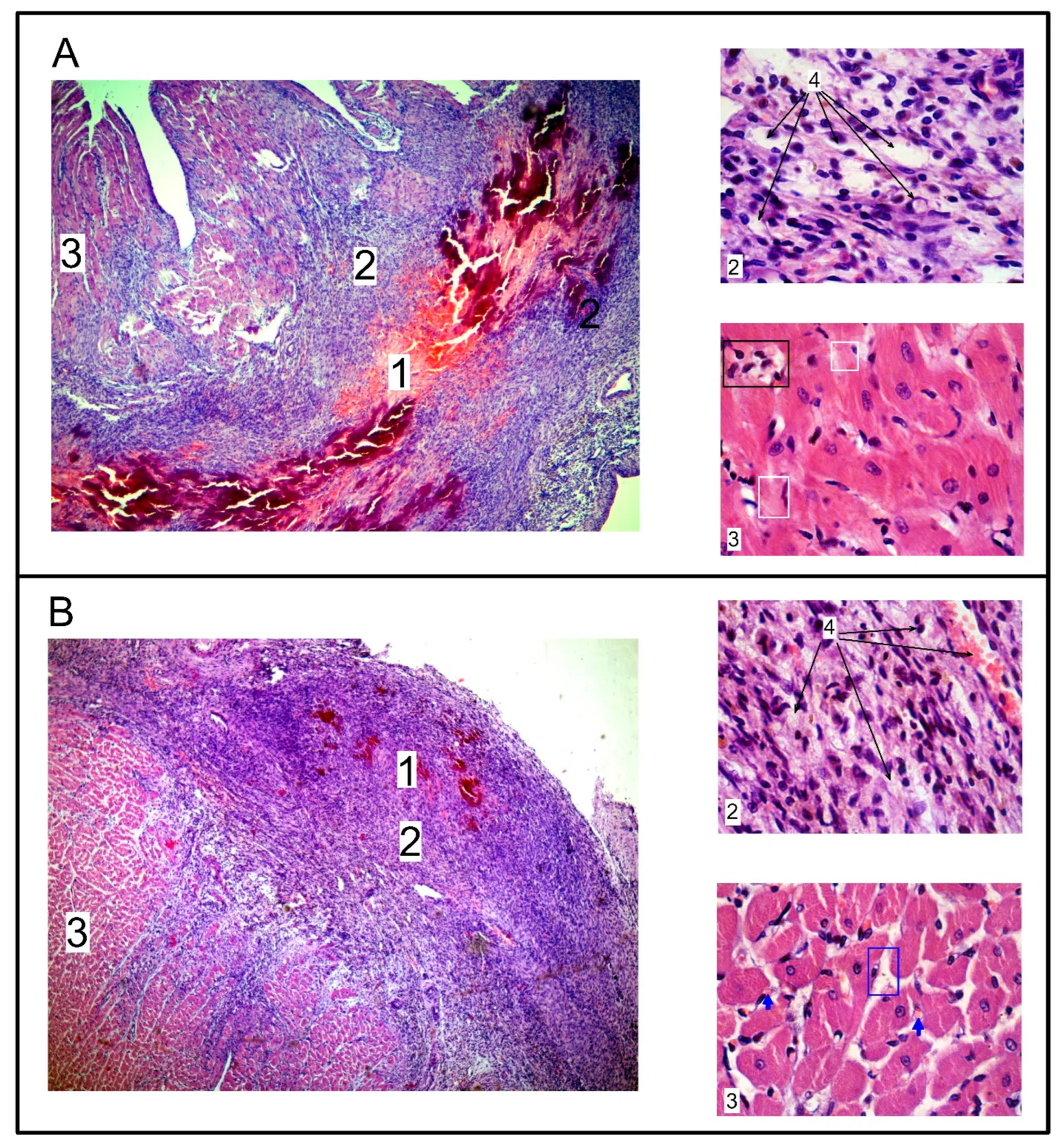
Rat myocardium after acute infarction on day 7 ((**A**)—OGD/Reperfusion; (**B**)—OGD/Reperfusion + toxin): an overview photograph (**A**) shows a necrotic focus occupying a large area of the section (1), including thrombotic masses and necrotic detritus. The zone of demarcation inflammation (2) occupies most of the section, represented by polymorphonuclear leukocytes, macrophages, fibroblasts, thin-walled vessels of different diameters, most of which are empty. The myocardium (3) is practically not expressed, characterized by inflammatory leukocyte infiltration; cardiomyocytes have hypertrophied nuclei and light-stained cytoplasm, individual cardiomyocytes with signs of myolysis (white frame—swollen cardiomyocytes, black frame—leukocyte infiltrate, blue frame—interstitial edema, blue arrows—blood-filled capillaries). In the overview photograph (**B**), a significant reduction in the size of the necrosis (1) is visible, compared to figure (**A**). Along the edges of the necrosis (2), formed granulation tissue is located, including polymorphonuclear leukocytes, fibroblasts, and collagen fibers. Zone B2 is distinguished by numerous blood vessels, predominantly filled with blood. Preserved myocardium (3) is characterized by edema, pronounced transverse striations, and capillaries filled with blood (arrows number 4—hemocapillaries in granulation tissue). Number of animals in the group: 3 days (n = 3), 7 days (n = 6 for control, n = 7 for toxin). Hematoxylin and eosin, *100, *600.

On the 7th day of the experiment, the infarction zone with pronounced eosinophilia is clearly visible in the control group of animals (Figure 7). Notably, there is early removal of necrotic debris and a decrease in the volume of necrotic masses in the myocardium of animals that received the toxin. The formation of granulation tissue ensures the replacement of the damaged area with a post-infarction connective tissue scar. Moreover, in animals that received the toxin, by the 7th day, there is no increase in the area of inflammation and no involvement of new areas of the myocardium in the affected area. The zone of demarcation inflammation in the perifocal areas of damage is replaced by foci of forming granulation tissue with numerous thin-walled blood vessels, both in animals of the experimental and control groups. However, the volume of granulation tissue in animals that received the toxin is higher on the 7th day compared to the animals of the control group, in which leukocyte infiltration of the stroma is still high. Macrophages are detected in the zone of inflammation of animals of all groups. The area of preserved myocardium is characterized by edema and hypertrophy of individual cardiomyocytes. In the myocardium of control animals, capillaries and larger vessels remain empty. In contrast, in the myocardium of animals injected with the toxin, the hemocapillaries contain red blood cells by day 7.

## 3. Discussion

Ischemia and subsequent reperfusion cause functional and metabolic disorders of the heart. Different types of ions are known to play a special role in the development of these disorders. The participation of Na+ ions in the development of IRI was shown as early as in the 90s of the XX century [4,20]. However, at present, few works are devoted to further study of the role of Na^+^ ions, as well as prevention of their influx at the stage of both ischemia and reperfusion. Despite this, several studies prove the hypothesis of the key role of Na^+^ in the development of IRI. For instance, propylparaben, an antimicrobial preservative, can significantly inhibit potential-dependent Na^+^-channels in a dose-dependent manner in isolated cardiomyocytes of adult rats, thus preventing the development of IRI [21].

Cariporide, an NHE (sodium/proton exchanger) inhibitor, also exerts a protective effect on cardiomyocytes during the development of ischemia/reperfusion injury by reducing Na+ concentration [16,22]. A cardiac potential-dependent sodium channel inhibitor, 3,4-dihydro-N-[(2S)-3-[(2-methoxyphenyl)thio]-2-methylpropyl]-2H-(3R)-1,5-benzoxathiepin-3-amine, has potent anti-ischemic effects in in vitro and in vivo models [23]. Ranolazine is a racemic mixture containing enantiomeric forms (S-ranolazine and R-ranolazine). In ventricular myocytes from dog and guinea pig hearts, it has been shown that ranolazine, at therapeutically relevant concentrations (up to 10 µM), selectively inhibits the late sodium current (late *I_N_*_a_) without affecting the rapid sodium current responsible for the upstroke phase of the action potential. Although the drug’s primary therapeutic use is the treatment of chronic stable angina, its unique mechanism of action makes it an effective agent for reducing tissue damage upon the restoration of blood flow. Administration of ranolazine (5 µM) over a period of 1 min immediately prior to 30 min of global ischemia reduced infarct size in isolated guinea pig hearts [14]. Another specific blocker of the late sodium current, F 15845, also exhibits a pronounced cardioprotective effect [15]. As research indicates, the beneficial role of sodium current inhibitors in the development of ischemia–reperfusion injury depends on the identification of highly selective agents capable of binding specifically to late voltage-gated sodium channels without affecting fast channels.

In this study, we propose using the peptide toxin mu-agatoxin-Aa1a as a cytoprotective agent; this toxin is capable of influencing sodium ion dynamics, as sodium is a key ion in the development of apoptosis. The strategy of utilizing natural peptide toxins that selectively bind to sodium ion channels has already been applied. For example, the use of tetrodotoxin (TTX), a sodium channel blocker, showed a significant increase in the number of viable cells in a model of isolated cardiomyocytes of rats [24]. In our previous study, when studying the effect of mu-agatoxin-Aa1a toxin on IRI in the culture of cells of epithelial origin, it was also shown that the toxin has a favorable effect on the survival of CHO-K1 culture primarily by maintaining the concentration of sodium and calcium ions at a preischemic level, as well as maintaining the pH level at an elevated level [25]. In the present study, the research was done on a culture of H9C2 embryonic cardiomyoblast cells capable of differentiating into cardiomyocytes. This culture is a relevant model for ischemia–reperfusion in vitro [26].

Cardiomyocyte death as a result of IRI occurs through both apoptosis and necrosis [26,27]. In the course of our study, we selected optimal conditions for the development of cell death during IRI modeling (16 h of OGD—oxygen-glucose deprivation and 2 h of reperfusion in complete nutrient medium). After IRI modeling in H9C2 culture, both apoptosis and necrosis develop, which is testified by the increase in LDH concentration (Figure 1A). Several studies have shown that activation of cardiomyocyte death mechanisms during IRI development is accompanied by changes in the intracellular concentration of Ca^2+^, Na^+^ and K^+^ ions. Thus, at the stage of ischemia there is an increase in Na^+^ ion concentration as a result of NHE activation and influx through membrane sodium channels. At the reperfusion stage, NHE activity increases due to the washout of acidic extracellular fluid and restoration of the proton gradient. Increased NHE activity leads to a secondary increase in Na^+^ concentration. To return to preischemic Na^+^ concentration there is an outflow of Na+ ions through the Na^+^/Ca^2+^ exchanger (NCH) at the expense of an influx of Ca^2+^ ions inside the cell. Ca^2+^ concentration increases, resulting in the activation of a cascade of reactions leading to cell death. On the other hand, increased Na+ concentrations cause water influx into the cell, resulting in swelling. Organelles such as mitochondria vacuolate, which interferes with cellular energy production and worsens fluid and electrolyte imbalances within the cell [28]. This can eventually lead to cell death when cellular integrity is lost. In this study, the development of cell death is accompanied by an increase in calcium and sodium ion concentration during the reperfusion stage (Figure 3). The addition of toxin at the reperfusion stage causes a decrease in the concentration of both Na^+^ and Ca^2+^ ions. However, in our study, we used undifferentiated cardiomyocytes. This culture consists of embryoblast-like myocytes and does not fully correspond to the morphology and phenotype of working cardiomyocytes, as it exhibits less pronounced voltage-gated ion channels. Consequently, no distinct change in potassium ion concentration was recorded—a factor that influences the overall conclusions—despite the observed responses in sodium and calcium ion levels.

In addition to changes in intracellular ion dynamics, the so-called “pH paradox” plays a key role in the development of IRI. Such a paradox has been well described. At the stage of ischemia in cardiomyocytes, acidosis develops (pH < or = 7.0); it provides a kind of protection of cells from ischemia, but after the stage of reperfusion, when the pH level returns to normal, the cells die; this is the paradox [29]. In our case, we see that in the first 10 min from the onset of reperfusion there is an increased pH level, which confirms the “paradox”. In the following minutes, pH decreases and then increases again, but in the OGD/Reperfusion group (Figure 3), pH increases significantly over the next 2 h of reperfusion, and increased cell death is also noted as a result of such a jump (Figure 1). The addition of toxin does not prevent a sharp increase in pH followed by a decrease in the first minutes of reperfusion, as in the OGD/Reperfusion group, but there is a smooth return of pH to normal after 2 h from the onset of reperfusion. A similar pattern was observed in a previous study with CHO-K1 culture: the toxin prevented a secondary sharp increase in pH, which also had an impact on cell viability.

While examining the effects of a toxin in an animal model of myocardial infarction, significant changes were also found. For example, the course of myocardial reparative regeneration in animals with non-reperfused myocardial infarction in the experimental groups on day 3 was characterized by an earlier onset of the organization stage and the formation of granulation tissue. On day 7, a single administration of the toxin resulted in more rapid removal of necrotic masses, early formation of granulation tissue, and the preservation and non-involvement of new myocardial tissue. Furthermore, a biochemical analysis of rat serum on day 3 post-MI modeling revealed that the activity of cardiac-specific enzymes, such as lactate dehydrogenase and creatine phosphokinase MB, which control energy metabolism in cardiomyocytes, reflects ischemic myocardial injury in all experimental groups. However, on the 7th day after injury, a significant decrease in LDH and CPK-MB was observed in the toxin-treated group of animals, compared to a marked increase in these parameters in the control group. The decrease in the activity of enzymes reflecting necrotic changes in the myocardium, LDH and CPK-MB, in the serum of rats in the toxin-treated group is consistent with the macroscopic analysis data assessing the risk/necrosis zone.

The development and maintenance of the action potential in the myocardium is regulated by the activity of numerous ion channel proteins. Transmembrane proteins provide a constant current of ions through ion channels to the sarcolemma of cardiomyocytes, which generate electrical impulses. The incoming current through sodium ion channels is the main driver of the excitatory cardiac impulse [30]. A possible mechanism of the protective action of the studied toxin is due to its modulating effect on fast sodium channels, which are responsible for rapid depolarization (phase 0 in both atrial and ventricular cardiomyocytes and Purkinje fibers) in the inactivated state. During myocardial infarction-induced ischemia, the toxin under study acts as an inhibitor of fast sodium channels, which causes a decrease in the action potential amplitude. As the membrane potential repolarizes during phase 3 of the cardiac cycle, the inactivated state of the channels prevents reactivation of the action potential, leading to refractoriness. Thus, due to the decrease in conductivity caused by the inhibition of sodium channels, the operation of ion re-entry mechanisms is interrupted, the duration of the action potential is changed, and an effective refractory period is created, which ultimately helps to stabilize the heart rhythm, preventing arrhythmias characteristic of myocardial infarction [31,32].

The study demonstrated the ability of mu-agatoxin-Aa1a to reduce cell damage during ischemia/reperfusion in vitro and in vivo experiments. These data suggest that mu-agatoxin-Aa1a enables myocardial cells to adapt to rapidly changing extracellular conditions upon restoration of normal blood flow after a period of ischemia. Mu-agatoxin-Aa1a binding to sodium channels prevents abrupt changes in intracellular ion concentrations and pH and, in our view, allows for a more gradual metabolic reorganization in response to normalized blood flow. In the absence of the toxin, intracellular metabolism can become disorganized due to a misalignment of the ion channel rhythm and the cellular energy supply with the orchestra of expressed genes. Although the primary focus regarding the toxin’s protective action under ischemia/reperfusion (I/R) conditions has been on sodium ion dynamics, future studies will investigate the toxin’s impact on intracellular signaling pathways, as the mechanism of action is not yet fully understood. In our study, the toxin’s efficacy under IRI conditions was demonstrated in an in vivo model; however, the effect was less pronounced in vitro, primarily due to the use of undifferentiated cell cultures. Nevertheless, the study allows us to regard mu-agatoxin-Aa1a as a biologically active molecule that specifically enhances cardiomyocyte adaptation to OGD/reperfusion conditions. It exhibits low toxicity, given its peptide nature and the fact that it is administered directly into the affected organ. This mode of action suggests that mu-agatoxin-Aa1a could serve as an adjunct therapy during balloon angioplasty.

## 4. Materials and Methods

### 4.1. Toxin Synthesis, Analysis, Purification and Folding

The toxin was obtained from SPPS on a ResPep SL peptide synthesizer (Intavis, Tübingen, Germany) using Fmoc-amino acids (Intavis, Tübingen, Germany) on TentaGel S RAM resin (Intavis, Tübingen, Germany) as previously described [33]. The toxin was analyzed using a Shimadzu LC-20AD XR HPLC system (Shimadzu, Kyoto, Japan) with an SPD-20A detector on a Dr. Maisch Luna C18 column. Protocol of gradient elution from 95% deionized water to 5% acetonitrile (Cryochrom, Saint Petersburg, Russia). MALDI-TOF MS FLEX series hardware and software system (Bruker Daltonics, Billerica, Massachusetts, USA) using the HCCA matrix (NPF Litech LLC, Moscow, Russia) was used for mass spectrometric analysis. HPLC (NGC Quest ™ 10 chromatography system (Bio-Rad, Hercules, CA, USA) was used for purification of toxin with Bio-Gel P-4 sorbent on an Econo-Column 1 cm × 30 cm column (Bio-Rad, Hercules, CA, USA) [34].

Folding buffer containing 10 mM reduced glutathione and 1 mM oxidized glutathione in 0.1 mol/L Tris-HCl (PanEco, Moscow, Russia), pH = 8.0, was used for folding and formation of the required secondary structure. Folding was carried out at 4 °C with gentle rocking for 24 h [35].

### 4.2. IRI Experimental Conditions for IRI Modeling

To model the conditions of cardiomyocyte IRI, we used H9C2 culture kindly provided by S.I. Shram from the Institute of Molecular Genetics of the Russian Academy of Sciences. Before the experiment, the culture was maintained in DMEM medium with 0.0045 kg/L glucose (Paneco, Moscow, Russia), 10% FBS (Globe Kang, Qinhuangdao, China), and penicillin/streptomycin mixture (Paneco, Moscow, Russia) in a CO_2_ incubator MCO-5AC (Sanyo, Osaka, Japan) at 37 °C and 5% CO_2_. Culture passages were performed every 7 days using 0.25% trypsin solution (Paneco, Moscow, Russia).

The experiment was performed in 96-well plates (SPL Life Sciences, Pocheon-si, Republic of Korea). Cells at a concentration of 5000 cells per well were left for 48 h for confluence. For IRI-like conditions modeling, the culture was placed in a CB-53 incubator (Binder, Tuttlingen, Germany) with 1% O_2_ in DMEM medium with 0.001 kg/L glucose and 1% FBS (oxygen-glucose-serum deprivation) for 16 h, then the plate was transferred to an MCO-5AC CO_2_ incubator with 5% CO_2_ and 18.6% O_2_ for 2 h [36]. Simultaneously, toxin at a final concentration of 50 nmol/L was added to the cells on the plate (the dose was determined experimentally using a dose–response curve and data from previous CHO-K1 culture studies [37]). The control group included cells incubated under normal conditions (18.6% O_2_, 5% CO_2_, DMEM with 0.0045 kg/L glucose and 10% FBS).

### 4.3. Detection of Basic Intracellular Parameters Using Fluorescent Dyes

Yo-Pro 1 (Invitrogen, Waltham, MA, USA) dye at a final concentration of 1 μmol/L [38] was used to analyze apoptosis levels. Dye at the final concentration was added to the cells at the end point of reperfusion (2 h) and incubated for 20 min at 37 °C in darkness in a CO_2_ incubator. The cells were then washed 2 times with 100 μL of warm PBS (pH 7.4) and left in 100 μL of fresh PBS for further imaging. Imaging was performed using CLARIOStar (BMG LabTech, Ortenberg, Germany) in matrix scanning mode with an automatically determined focal height at wavelengths characteristic of each dye. The acquired data were then processed in Excel and counted per 100,000 cells. Cell concentration was determined using a Goryaev chamber. Data were presented in the ratio of experiment measurements to control ones.

The concentration of calcium, sodium and potassium ions was detected at the end point of reperfusion (2 h): Rhod 2 AM (Abcam, Cambridge, UK) at a final concentration of 500 nmol/L [39] was used to determine Ca^2+^ concentrations, ION NATRIUM Green 2 AM (Abcam, Cambridge, UK) 500 nmol/L [40] for Na^+^, and for K^+^-ION Potassium Green 2 AM (Abcam, Cambridge, UK) 500 nmol/L [41]. After staining, the cells were incubated three times in warm PBS to wash off unbound dye. After this, the fluorescence signal was recorded using an optical system equipped with a Nikon Ti-S microscope, DS-Qi1MC camera, S Plan Fluor ELWD 20× 0.45 objective lenses, 465–495/515–555 nm filter (Nikon, Minato, Tokyo, Japan), and a computer with NIS-elements 4.0 software package. The quantitative analysis of images was performed with an ImageJ program (v. 1.45s). The fluorescence signal from more than 300 cells was analyzed for each group. The fluorescence intensity was calculated by the following formula: total cell fluorescence = integral density − (area of the selected cell × mean fluorescence of the background indicators).

To determine pH, 1 μmol/L BCECF AM (Servicebio, Wuhan, China) dye [42] was added to the cell at different time points (10, 40, and 120 min) and incubated for 20 min in the dark. The manipulations described above were then repeated, and imaging was performed in CLARIOStar at two wavelengths (Ex 490 and 430 (isobestic point), Em 535). The ratio of Ex/Em 490/535 nm and 430/535 was used to control staining.

### 4.4. Determination of LDH Concentration

The LDH Abris+ kit (Abris, Saint Petersburg, Russia) and a Genesys 10S UV-VIS spectrophotometer (Thermo Fisher Scientific, Waltham, MA, USA) were used to determine LDH concentration. The analysis was carried out as described in the article [43]. The percentage of cytotoxicity was determined using the formula:

$$\%\;cytotoxicity=\frac{Experimental\;LDH}{Total\;LDH}$$

where experimental LDH—concentration of LDH in the medium after the experiment with deduction of LDH in pure medium, total LDH—concentration of LDH in normal conditions taking account of conversion to cell concentration with deduction of LDH in pure medium.

### 4.5. Statistical Data Processing for In Vitro

The in vitro study was conducted in three replicates, with three groups per replicate. For the fluorescence microscopy data analysis, 300 cells were sampled per replicate. The asymmetry and kurtosis criteria were used to determine the nature of the distribution. To assess the statistical significance of differences, the Mann–Whitney test was used (due to a small sample size); processing was performed in the Origin software v. 95E (OriginLab Corporation, Northampton, MA, USA). Since the toxin exposure data were compared with the control conditions without a toxin, as well as with normal conditions, the Bonferroni test was used to eliminate the effect of multiple comparisons; differences between groups were considered statistically significant at *p* ≤ 0.02.

### 4.6. Ischemia–Reperfusion Injury Modeling

Myocardial ischemia followed by reperfusion was modeled using 20 male Wistar rats (SPF status, bred at the Laboratory Animal Nursery, Branch of the Institute of Bioorganic Chemistry, Russian Academy of Sciences, Pushchino) weighing 250–320 g in accordance with the relevant guidelines and regulations and the protocol. The study was approved by the Institutional Animal Care and Use Committee (IACUC) of the Almazov National Medical Research Center of the Russian Ministry of Health on 13 November 2024. The animals were randomly assigned to the following groups: 3 animals each in the control and toxin groups sacrificed on day 3 of the experiment, and 7 animals each in the groups sacrificed on day 7. The animals’ condition was assessed upon arrival at the animal facility, at the time of randomization, and immediately prior to the study. Assessments were performed by a veterinarian in accordance with standard examination procedures. Any signs of distress (e.g., social withdrawal, indications of pain) were grounds for excluding an animal from the experiment and transferring it to an acclimatization room pending a decision on its suitability for the study. Exclusion criteria: MAP < 50 mmHg and/or HR < 300 at any time during the experiment (one animal was excluded from the control group due to death during the induction of anesthesia (respiratory arrest)). After the exclusion of one animal, the group distribution was as follows: day 3: 6 animals (*n* = 3 control, *n* = 3 toxin); day 7: 13 animals (*n* = 6 control, *n* = 7 toxin).

The study utilized a model of regional myocardial ischemia–reperfusion in rats in vivo. Rats were anesthetized with isoflurane IsoNic (Vetpharma Animal Health, Barcelona, Spain). After anesthesia, the rats were placed on mechanical ventilation (CWE-SAR-830/AP, World Precision Instruments, Inc., FL, USA) with a preset respiratory rate of 60 breaths per minute (ATC1000-220, World Precision Instruments, Inc., FL, USA). The isoflurane concentration was adjusted within the 4–5% range, guided by arterial blood pressure and heart rate readings. During the surgery and the subsequent observation period, the animals were placed on a table with a thermostatically controlled surface maintained at 37 °C to prevent hypothermia.

To induce regional ischemia–reperfusion injury, the chest was opened by an incision in the fourth intercostal space. The ribs were spread to expose the heart, then the pericardium was opened, and a 6.0 polypropylene ligature with an atraumatic needle was inserted under the main branch of the left coronary artery, approximately 2 mm from its origin [44]. The ends of the ligature were passed through an occluder—a small polyethylene tube ~7–8 cm (PE-90) (BD, New Jersey, U.S.) and brought out. Reversible myocardial ischemia was created by sliding the occluder down the ligatures and applying a surgical clamp to the occluder to prevent its backward displacement. After completion of the surgical procedures and a 30 min stabilization period, myocardial ischemia was initiated. Ischemia lasted 30 min with recording of ECG parameters and monitoring of ST-segment elevation. Reperfusion was initiated after 30 min by removing the ligature. Jugular vein catheterization was performed for infusion of the test toxin, which was administered immediately at the onset of reperfusion in a volume of 100 μL at a rate of 0.36 μg/g (the dose was determined based on data from culture experiments, considering the administration of the drug directly into the affected area (jugular vein)). The parameters were recorded over the subsequent 60 min.

During the postoperative period, pain management was achieved by administering daily injections of Meloxicam at a dose of 2 mg/kg, subsequently reduced to 1 mg/kg until the end of the experiment. Euthanasia of animals on the 3rd and 7th days was performed by an overdose of isoflurane anesthesia (more than 5%) followed by collection of venous blood and organs.

### 4.7. Identification of the Risk Zone and Necrosis Zone

At the end of the experiment, after the animal was euthanized, the heart was removed and perfused for several minutes with 2.5 mL of 2.0% Evans Blue (MP Biomedicals, Santa Ana, CA, USA). Hearts were excised and cut into five 2 mm-thick slices parallel to the atrioventricular groove. The basal surface of each slice was photographed using a digital camera. The slices were immersed in 1% 2,3,5-triphenyltetrazolium chloride (TTC) (ICN Pharmaceuticals, California, USA) at 37 °C (pH 7.4) for 15 min and photographed again to identify the infarct area and record the fluorescence area and ICG intensity. Images were analyzed using ImageJ (v. 1.45s). The area at risk (Evans-negative areas) and the area of myocardial necrosis (TTC-negative areas) were expressed as a percentage of the entire slice. The area at risk and infarction area values for each heart were obtained by summing the data for the slices and calculating the mean values [17].

### 4.8. Histological Analysis

Experimental material was fixed in 10% neutral formalin (histological formalin, manufactured by Labiko LLC, Russia) for 7 days. For morphological analysis, staining was performed with Carracci hematoxylin and eosin according to the standard prescription, using alcohols of 80 and 96% concentrations, alcohol-xylene, xylene (Ortho-xylene, manufactured by Labiko LLC, Moscow, Russia), Carracci hematoxylin (Labiko LLC, Moscow, Russia), 1% aqueous eosin (Labiko LLC, Moscow, Russia). Stained slices on glass slides were covered with a cover slip (24 mm × 24 mm, 24 mm × 32 mm or 24 mm × 40 mm, ApexLab, Yancheng, China) using a mounting medium Glasseal (Labiko LLC, Moscow, Russia). Description and comparative morphological analysis of permanent micro-preparations were carried out using a video test system, including a binocular microscope “Micromed 3 (Professional)” (Micromed, Shenzhen, China) and a video eyepiece “ToupCam UCMOS” (Hangzhou ToupTek Photonics Co., Ltd., Hangzhou, China) at magnifications of 10 × 16, 10 × 40, 10 × 60, 10 × 100.

### 4.9. Blood Biochemistry Test

Venous blood was collected from the tail vein before organ retrieval. Blood biochemistry tests were performed on an ERBA-XL-100 biochemistry analyzer (Erba Lachema s.r.o, Brno, Czech Republic) using the manufacturer’s kits and calibrators. Blood samples were analyzed in the laboratories of the Medical and biological center of the S.P. Kapitsa Research Institute of Technology at Ulyanovsk State University.

### 4.10. Statistical Analysis for In Vivo

The obtained primary data were tested for normality of the distribution of the studied parameter values (Shapiro–Wilk, W-test). If the values were normally distributed, comparisons of intergroup values were performed using Student’s *t*-test (at a 5% significance level). If the data were not normally distributed, comparisons of intergroup values were performed using the nonparametric Mann–Whitney analysis (at a 5% significance level) (LDH level).

Primary statistical analysis for the in vivo study included descriptive statistics of quantitative parameters. The number of animals was: day 3: 6 animals (*n* = 3 control, *n* = 3 toxin); day 7: 13 animals (*n* = 6 control, *n* = 7 toxin). The arithmetic mean (M), standard deviation (±σ), and significance of the difference in arithmetic means (*p* < 0.05) were calculated using Student’s *t*-test or the Mann–Whitney U-test (*p* < 0.05). Risk zones and histological sections were evaluated using a single-blind method. Statistical analysis was performed using Statistica 6.0 (USA) and StatTech v. 3.1.10 (developer—Stattech LLC, Kazan, Russia).

## Data Availability

The datasets generated during and/or analyzed during the current study are available from the corresponding author on reasonable request.

## References

[1] Saleh M., Ambrose J.A. (2018). Understanding myocardial infarction. F1000Research.

[2] Murphy E., Steenbergen C. (2008). Mechanisms underlying acute protection from cardiac ischemia-reperfusion injury. Physiol. Rev..

[3] Simonis G., Strasser R.H., Ebner B. (2012). Reperfusion injury in acute myocardial infarction. Crit. Care.

[4] Pie G., Czubryt M. (1995). The contribution of ionic contribution of ionic imbalance to ischemia/reperfusion-induced injury. J. Mol. Cell. Cardiol..

[5] Sanada S., Komuro I., Kitakaze M. (2011). Pathophysiology of myocardial reperfusion injury: Preconditioning, postconditioning, and translational aspects of protective measures. Am. J. Physiol. Heart Circ. Physiol..

[6] Garcia-Dorado D., Ruiz-Meana M., Inserte J., Rodriguez-Sinovas A., Piper H.M. (2012). Calcium-mediated cell death during myocardial reperfusion. Cardiovasc. Res..

[7] Persson F., Andersson B., Duker G., Jacobson I., Carlsson L. (2007). Functional effects of the late sodium current inhibition by AZD7009 and lidocaine in rabbit isolated atrial and ventricular tissue and Purkinje fibre. Eur. J. Pharmacol..

[8] Fearon I.M., Brown S.T. (2004). Acute and chronic hypoxic regulation of recombinant hNav1.5 α subunits. Biochem. Biophys. Res. Commun..

[9] Deshmukh A., Larson J., Ghannam M., Saeed M., Cunnane R., Ghanbari H., Latchamsetty R., Crawford T., Jongnarangsin K., Pelosi F. (2021). Efficacy and tolerability of quinidine as salvage therapy for monomorphic ventricular tachycardia in patients with structural heart disease. J. Cardiovasc. Electrophysiol..

[10] Kelson K., de Souza I. (2019). Procainamide versus amiodarone for stable ventricular tachycardia. Acad. Emerg. Med..

[11] Andrikopoulos G.K. (2015). Flecainide: Current status and perspectives in arrhythmia management. World J. Cardiol..

[12] Nakamura T., Kashimoto S., Oguchi T., Kumazawa T. (1992). Effects of propafenone on function and metabolism in the ischemic working rat heart. Heart Vessel..

[13] Brunsting L.A., Jessen M.E., Abd-Elfattah A.S., Mask W.K., Godwin C.K., Wechsler A.S. (1987). Myocardial protective effects of the class Ic antiarrhythmic agent flecainide. J. Thorac. Cardiovasc. Surg..

[14] Aldakkak M., Camara A.K., Heisner J.S., Yang M., Stowe D.F. (2011). Ranolazine reduces Ca2+ overload and oxidative stress and improves mitochondrial integrity to protect against ischemia reperfusion injury in isolated hearts. Pharmacol. Res..

[15] Létienne R., Bel L., Bessac A.M., Vacher B., Le Grand B. (2009). Myocardial protection by F 15845, a persistent sodium current blocker, in an ischemia-reperfusion model in the pig. Eur. J. Pharmacol..

[16] Metwally S.A.H., Paruchuri S.S., Yu L., Capuk O., Pennock N., Sun D., Song S. (2023). Pharmacological Inhibition of NHE1 Protein Increases White Matter Resilience and Neurofunctional Recovery after Ischemic Stroke. Int. J. Mol. Sci..

[17] Sonin D., Medved M.S., Khapchaev A.Y., Sidorova M.V., Palkeeva M.E., Kazakova O.A., Papayan G.V., Mochalov D.A., Minasyan S.M., Anufriev I.E. (2025). Antiedemic Effect of the Myosin Light Chain Kinase Inhibitor PIK7 in the Rat Model of Myocardial Ischemia Reperfusion Injury. Curr. Issues Mol. Biol..

[18] dos Santos W.F. (2009). Spider and wasp neurotoxins. Botulinum Toxin.

[19] Obrenovitch T.P. (1996). Chapter 6 Sodium and Potassium Channel Modulators: Their Role in Neuroprotection. Int. Rev. Neurobiol..

[20] Takeo S., Tanonaka K., Liu J.-X., Kamiyama T., Ohoi I., Kajiwara H., Kameda H. (1998). Ischemia/reperfusion injury and ion channel blockade. Protection Against Ischemia/Reperfusion Damage of the Heart.

[21] Ji X., Xu Z., Criswell H.E., Boysen P.G. (2004). Propyl paraben inhibits voltage-dependent sodium channels and protects cardiomyocytes from ischemia-reperfusion injury. Life Sci..

[22] Baetz D., Bernard M., Pinet C., Tamareille S., Chattou S., El Banani H., Coulombe A. (2003). Different pathways for sodium entry in cardiac cells during ischemia and early reperfusion. Mol. Cell. Biochem..

[23] Le Grand B., Pignier C., Létienne R., Cuisiat F., Rolland F., Mas A., Vacher B. (2008). Sodium late current blockers in ischemia reperfusion: Is the bullet magic?. J. Med. Chem..

[24] Yang C.-K., Yang S.-Q., Tan S.-T. (2009). Effects of cardioplegia with tetrodotoxin on intracellular sodium overload of ischemia/reperfusion cardiomyocytes. Zhongguo Ying Yong Sheng Li Xue Za Zhi.

[25] Son E., Lee D., Woo C.-W., Kim Y.-H. (2020). The optimal model of reperfusion injury in vitro using H9c2 transformed cardiac myoblasts. Korean J. Physiol. Pharmacol..

[26] Krijnen P.A.J. (2002). Apoptosis in myocardial ischemia and infarction. J. Clin. Pathol..

[27] Tavernarakis N. (2007). Cardiomyocyte necrosis: Alternative mechanisms, effective interventions. Biochim. Biophys. Acta Mol. Cell Res..

[28] Pedriali G., Ramaccini D., Bouhamida E. (2022). Perspectives on mitochondrial relevance in cardiac ischemia/reperfusion injury. Front. Cell Dev. Biol..

[29] Bond J.M., Chacon E., Herman B., Lemasters J.J. (1993). Intracellular pH and Ca2+ homeostasis in the pH paradox of reperfusion injury to neonatal rat cardiac myocytes. Am. J. Physiol. Cell Physiol..

[30] Bokeria O.L., Akhobekov A.A. (2024). Ion channels and their role in the development of arrhythmias. Non-Invasive Arrhythmology.

[31] Chaudhary K.W. (2024). An overview of drug-induced sodium channel blockade and changes in cardiac conduction: Implications for drug safety. Clin. Transl. Sci..

[32] Lu H.R., Rohrbacher J., Vlaminckx E., Van Ammel K., Yan G.X., Gallacher D.J. (2010). Predicting drug-induced slowing of conduction and pro-arrhythmia: Identifying the ’bad’ sodium current blockers. Br. J. Pharmacol..

[33] Iurova E., Rastorgueva E., Beloborodov E., Pogodina E., Fomin A., Sugak D., Viktorov D., Tumozov I., Saenko Y. (2023). Protective Effect of Peptide Calcium Channel Blocker Omega-Hexatoxin-Hv1a on Epithelial Cell during Ischemia-Reperfusion Injury. Pharmaceuticals.

[34] Mant C.T., Chen Y., Yan Z., Popa T.V., Kovacs J.M., Mills J.B., Tripet B.P., Hodges R.S. (2007). HPLC analysis and purification of peptides. Methods Mol. Biol..

[35] Moore S.J., Leung C.L., Norton H.K., Cochran J.R. (2013). Engineering agatoxin, a cystine-knot peptide from spider venom, as a molecular probe for in vivo tumor imaging. PLoS ONE.

[36] Wenger R.H., Kurtcuoglu V., Scholz. C.C., Marti H.H., Hoogewijs D. (2015). Frequently asked questions in hypoxia research. Hypoxia.

[37] Iurova E., Beloborodov E., Rastorgueva E., Fomin A., Saenko Y. (2023). Peptide sodium channels modulator mu-agatoxin-Aa1a prevents ischemia-reperfusion injury of cells. Molecules.

[38] Gallardo Bolaños J.M., Miró Morán Á., Balao da Silva C.M., Morillo Rodríguez A., Plaza Dávila M., Aparicio I.M., Tapia J.A., Ortega Ferrusola C., Peña F.J. (2012). Autophagy and apoptosis have a role in the survival or death of stallion spermatozoa during conservation in refrigeration. PLoS ONE.

[39] Fonteriz R.I., de la Fuente S., Moreno A., Lobatón C.D., Montero M., Alvarez J. (2010). Monitoring mitochondrial [Ca2+] dynamics with rhod-2, ratiometric pericam and aequorin. Cell Calcium.

[40] Tay B., Stewart T.A., Davis F.M., Deuis J.R., Vetter I. (2019). Development of a high-throughput fluorescent no-wash sodium influx assay. PLoS ONE.

[41] Camilli G., Bohm M., Piffer A.C., Lavenir R., Williams D.L., Neven B., Grateau G., Georgin-Lavialle S., Quintin J. (2020). β-Glucan–induced reprogramming of human macrophages inhibits NLRP3 inflammasome activation in cryopyrinopathies. J. Clin. Invest..

[42] Alvarez-Leefmans F.J., Herrera-Pérez J.J., Márquez M.S., Blanco V.M. (2006). Simultaneous measurement of water volume and pH in single cells using BCECF and fluorescence imaging microscopy. Biophys. J..

[43] Kumar P., Nagarajan A., Uchil P.D. (2018). Analysis of cell viability by the lactate dehydrogenase assay. Cold Spring Harb. Protoc..

[44] Himori N., Matsuura A. (1989). A simple technique for occlusion and reperfusion of coronary artery in conscious rats. Am. J. Physiol..

